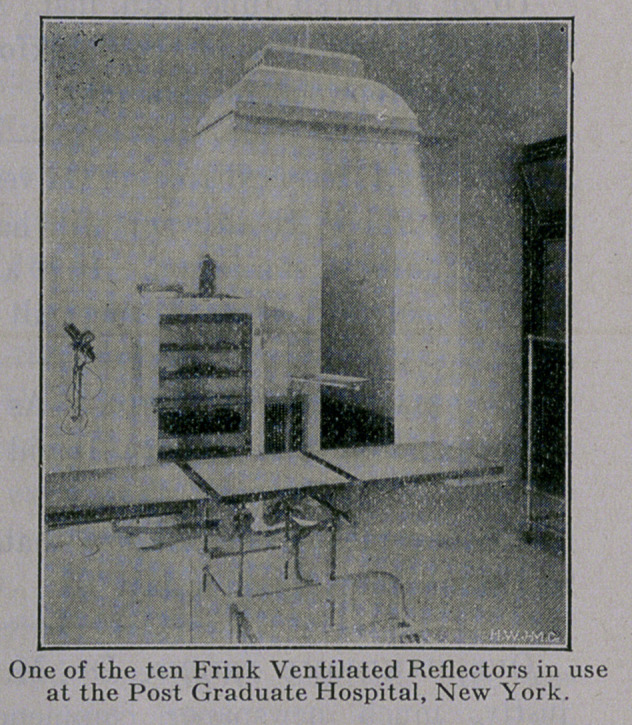# Proper Illumination of the Operating Table

**Published:** 1913-06

**Authors:** 


### Abstracts and Selections.

### Proper Illumination of the Operating Table.

Hospital authorities have, in the past, experienced considerable difficulty in obtaining proper illumination for the operating table. Surgeons have frequently been obliged to perform delicate operations with insufficient light on the patient, and in many cases the surgeon's head has been subjected to an intense heat caused by radiation.

Working in this manner, under poor lighting conditions, and at the same time suffering positive discomfort, it is not surprising that surgeons should enthusiastically indorse the Frink Ventilated Operating Reflector.

This reflector is designed on scientific lines and is the product of long and careful study to produce an illuminating device which will give the proper quality, intensity and distribution of light—with a minimum of heat. So brilliant is the illumination pro-

Frink Conical Microscopic Reflector.

One of the ten Frink Ventilated Reflectors in use at the Post Graduate Hospital, New York.

duced by a Frink Reflector that every minute detail of the body is shown in perfect clearness.

The reflector proper is made of white enameled metal, designed for six 100-watt Tungsten lamps. Its useful illumination is increased *eight-fold* by the use of silvered ripple glass. By this scientific method an intensity of forty-three foot candles is produced on the working plane. No other known device gives such a brilliant, white light and concentrates it so effectively upon an operating table.

An equally remarkable feature of the Frink Reflector is its ingenious ventilating arrangement. It would be impracticable for a surgeon to operate under a working intensity of forty-three foot-candles if the heat were not automatically carried off. The patented Frink construction provides for this in the following manner:

Two tubes or funnels are mounted vertically inside the reflector. When these funnels become heated a partial vacuum is created within them. Cool air is drawn in between the two glass plates, while the heated air is expelled through the funnels. The ingenious arrangement of these two plates and the funnels insure a constant circulation. The natural result is a bank of cool air above the head of the operating surgeon, enabling him to work without discomfort. Careful tests have shown only eight degrees variation of temperature after an hour's continuous use.

Frink Ventilated Operating Table Reflectors are made in stationary form, with adjustable fittings, or with a raising and lowering device. Some are arranged for eight 35-watt J-M Linolite Lamps. Special apparatus is supplied when desired.

Scores of hospitals throughout the country are now successfully using this system of illumination. In New York City alone Frink Operating Reflectors are installed in the Bellevue, Post Graduate, Sea View, East Tuberculosis, Metropolitan, Gouveneur, Mt. Sinai, German, Emergency and City Hospitals.

The Frink System of Lighting also includes Dustless Ward Reflectors, Adjustable Bed Lights, Floor Lights, Therapeutic Reflectors, Reflectors for Microscopic Work, etc.

The H. W. Johns-Manville Co., New York, Sole Selling Agents, will be glad to send their illustrated Catalog, "Modern Hospital Illumination," to anyone interested in hospital illumination.

---

Loening, of Halle (*Munch. med. Woch.* 1912, Nos. 9, 10 and 11), says: "Melubrin behaves as a specific in acute articular rheumatism. The remedy can even be used if endocarditis is present. It also gives positive results in chronic articular rheumatism, myositis and sciatica. I have given over 45,000 grains of melubrin and have seen no failures."

Schrenk, of the University of Heidelberg (*Deutsch. med. Woch.* 1912, No. 34), after using 2,500 grams on 60 patients, reports that, "We were able to confirm the observations of Loening that one can cure articular rheumatism without salicylic acid preparations. We hold this to represent a very essential advance in medicine, for not every patient can tolerate salicylic acid, and a substitute then becomes absolutely necessary."

Prof. Edward Martin, of the University of Pennsylvania, writing on the treatment of syphilis in the latest volume of Keen's *Surgery*, Vol. VI, p. 111, says: "Salvarsan is indicated in all stages of syphilis. Given in full dosage and repeated in seven days, it produces its maximum safe effect, and if used in the early stage of chancre, seems capable of producing an immediate and permanent cure. The lesions of syphilis yield more promptly to salvarsan than to mercury, and the Wassermann reaction becomes negative in a larger proportion of cases. It is generally accepted that salvarsan should be supplemented by mercury, the latter being given in doses as large as are campatible with bodily and mental vigor, preservation of appetite and digestion, free elimination, and the holding of the normal weight."

## Figures and Tables

**Figure f1:**
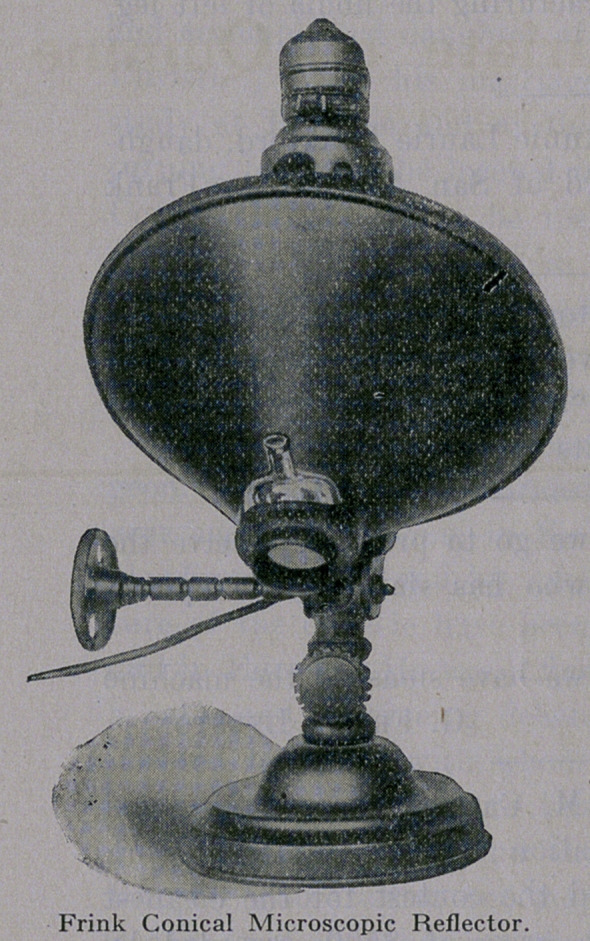


**Figure f2:**